# Regional Aquifer Vulnerability and Pollution Sensitivity Analysis of Drastic Application to Dahomey Basin of Nigeria

**DOI:** 10.3390/ijerph17072609

**Published:** 2020-04-10

**Authors:** Saheed Adeyinka Oke

**Affiliations:** Civil Engineering Department, Central University of Technology Free State, Bloemfontein 9301, South Africa; soke@cut.ac.za or okesaheed@gmail.com

**Keywords:** Dahomey Basin, DRASTIC, groundwater, pollution sensitivity analysis, vulnerability mapping

## Abstract

Shallow groundwater vulnerability mapping of the southwestern Nigeria sedimentary basin was assessed in this study with the aim of developing a regional-based vulnerability map for the area based on assessing the intrinsic ability of the aquifer overlying beds to filter and degrade migrating pollutant. The mapping includes using the established seven parameter-based DRASTIC vulnerability methodology. Furthermore, the developed vulnerability map was subjected to sensitivity analysis as a validation approach. This approach includes single-parameter sensitivity, map removal sensitivity, and DRASTIC parameter correlation analysis. Of the Dahomey Basin, 21% was classified as high-vulnerability and at risk of pollution, 61% as moderate vulnerability, and 18% as low vulnerability. Low vulnerability areas of the basin are characterised by thick vadose zones, low precipitation, compacted soils, high slopes, and high depth to groundwater. High-vulnerability areas which are prone to pollution are regions closer to the coast with flat slopes and frequent precipitation. Sensitivity of the vulnerability map show the greatest impact with the removal of topography, soil media, and depth to groundwater and least impact with the removal of the vadose zone. Due to the subjectivity of the DRASTIC method, the most important single parameter affecting the rating system of the Dahomey Basin DRASTIC map is the impact of the vadose zone, followed by the net recharge and hydraulic conductivity. The DRASTIC vulnerability map can be useful in planning and siting activities that generate pollutants (e.g., landfill, soak away, automobile workshops, and petrochemical industries) which pollute the environment, groundwater, and eventually impact the environmental health of the Dahomey Basin’s inhabitants.

## 1. Introduction

The reliance on groundwater by many third world countries is due to many reasons, including its low susceptibility to surface pollution compared to surface water (rivers, lakes, and springs). In many rural areas of developing countries, groundwater serves as the mainstay for domestic and agricultural irrigation. The relatively low cost of developing groundwater for drinking purposes in many African communities suggests reasons why groundwater is dependably relied on for quality water supply over other water sources [1]. The dependency of groundwater for drinking purposes has not reduced its mismanagement and pollution, which has great health implications. One of the best ways of minimizing groundwater pollution is the mapping of groundwater vulnerability. Mapping of intrinsic parameters above an aquifer can predict the likely consequences of surface pollutant loading [2] that eventually pollute groundwater resources. Vulnerability is not an absolute characteristic, but rather a relative, non-measurable, dimensionless property, indicating where contamination is most likely to occur [3] and to impact water quality. Groundwater vulnerability mapping can provide valuable information for stakeholders working on preventing further deterioration of the environment and pollution migration to groundwater resources, especially if such pollution might affect the environmental health of the community [4].

Several vulnerability approaches have been developed since the first usage of the term “groundwater vulnerability” by [5]. Some of the common approaches are GOD [6], AVI [7], SINTACS [8], RTt [9], and EPIK [10]. The DRASTIC approach, developed by Aller et al. [11], is one of the most widely used methods [12,13,14,15,16,17,18,19,20]. DRASTIC was developed for the United States Environmental Protection Agency (USEPA), with the purpose of creating a methodology that would permit a systematic evaluation of the potential groundwater pollution of any hydrogeological setting [3]. The method is based on the parametric system method that weights critical factors affecting vulnerability, and is also based on the Delphi technique [11]. The advantages of the DRASTIC approach include its straightforward application, simplicity, and regional applicability. However, its subjectivity of assigning values to parameters has been critically questioned [21,22].

Groundwater vulnerability mapping of aquifers in developing countries must be prioritized, particularly in areas characterised by a low level of environmental sanitation awareness, adherence to urban planning, and environmental health of the inhabitance. In many developing countries of the world, as well as in the study area, statewide groundwater vulnerability maps are still undergoing development or are rarely attempted [23,24]. Therefore, this paper has been aimed at developing a regional-based vulnerability map of the Dahomey Basin of Nigeria’s shallow aquifers, using the DRASTIC-based parameters which will serve as a background for pollution migration prediction and associated health risks that might have an eventual impact. Regional aquifers are important for protection and considered more valuable than less productive or poor aquifers [25]. 

The main purpose of assessing the intrinsic vulnerability of the shallow aquifer is to predict the risk associated with such an aquifer [26] and the capacity of the overlying beds serving as a filter of contaminants released from the surface. Examples of these contaminants are nitrate from pit latrines, heavy metals from non-engineered landfills, trace metals from automobile workshops, and microbial loadings. Therefore, an aquifer will pose higher health risks if it has poor intrinsic vulnerability potential due to inability of the overlying bed or the aquifer materials to filter and degrade transporting contaminants. Hence, DRASTIC vulnerability assessment is the most cost-effective and less time-consuming method that can be applied to assess the regional shallow groundwater vulnerability of an area, such as the Dahomey Basin, where uncontrolled development occurs with land activities. DRASTIC is preferred for assessing regional aquifers compared to assessment of specific sites or contaminated aquifers [27]. A few studies on regional-based assessment with the DRASTIC methodology similar to the study area include assessment of the Barka region of Oman [28], groundwater mapping of Portugal [29], aquifer vulnerability assessment of the Paluxy aquifer, Central Texas, USA [30], and assessment of groundwater in the Melaka State of Malaysia [31]. 

### 1.1. Study Area

**Figure 1:**
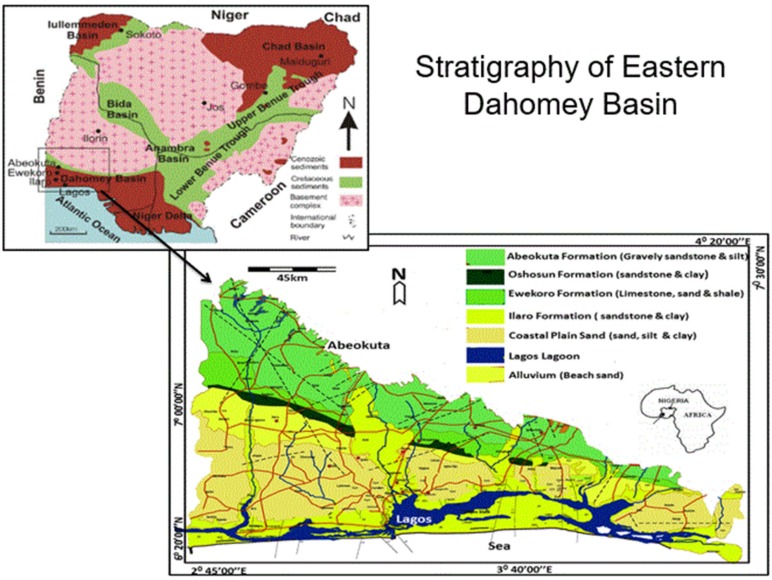
Map of Dahomey Basin showing rock types, stratigraphy succession, and location within Nigeria.

Shallow groundwater of the Dahomey Basin of southwestern Nigeria was targeted for the vulnerability mapping, because large human population settlement depends on this coastal sedimentary aquifer for domestic and industrial purposes. This settlement includes Lagos, the world’s sixth largest city and Africa’s most populous city. The estimated density of 2607 people per square kilometre lived in Lagos in 2006 [32], and it is projected to become 5032 people per square kilometer in 2025 [33]. Lagos’ shallow groundwater is undergoing increased potential for contamination due to this remarkable growth in population, and industrial and agricultural activities. The average annual rainfall for the Dahomey Basin is 1800 mm for Lagos and areas along the coast, and 1200 mm for Abeokuta city at the northern end of the Basin. The temperature fluctuates between 25–30 °C annually. The most important surface water flowing in the Basin is that of the Yelwa River and the Ogun River, as well as its numerous tributaries, including the Ewekoro River. The Dahomey Basin is a transboundary basin extending into Ghana [34]. The eastern half of the basin is situated in southwestern Nigeria (Figure 1). The geology of the basin is composed of sedimentary formations of Late Cretaceous and Tertiary ages [35]. Rock types include limestone, clay, shale, massive sandstone, alluvium, and unconsolidated sand and silt [36]. 

### 1.2. Hydrogeology

Common means of accessing groundwater in the Dahomey Basin include construction of large-diameter, hand-dug wells and tube wells. This is partly due to the shallow nature of the unconfined aquifers and the predominantly sedimentary formations of alluviums, limestones, and sandstones with intercalated clay rock types. The basin hydrology has been outlined by Offodile [37] who showed that the most important hydro-stratigraphical unit in the basin for shallow aquifer mapping are the recent alluvium and the Coastal Plain Sand aquifers in Lagos and environs, cretaceous aquifer of the Abeokuta Formation in Ijebu-Ode and Abeokuta areas, Oshosun/Ilaro Formation aquifers in Shagamu and Ilaro axes, and the Ewekoro Formations aquifers in the Ibeshe, Igbogilla, and Ewekoro areas (Figure 1). Towards the coast, the three main hydro-stratigraphic units are the Upper Aquifer (Alluvium and Coastal Plain Sands), the Middle Aquifer (Ilaro and Ewekoro Formations), and the Lower Aquifer (Abeokuta Formations) [38]. The Basement Complex rock of southwestern Nigeria underlies most Abeokuta Formation aquifers in the northern areas of the Dahomey Basin. Adelana et al. [1] reported the pressure on the groundwater resources of Lagos, and about 75% of groundwater abstracted in Lagos for domestic and industrial purposes is obtained from the Coastal Plain Sand aquifer.

## 2. Materials and Methods

The DRASTIC method is based on the following seven parameters: depth to groundwater (D), net recharge (R), aquifer media (A), soil media (S), topography (T), vadose zone impact (I), and hydraulic conductivity (C). The source of data used in the computation of the DRASTIC method and the description of the factors considered in the assessment are shown in Table 1. For the DRASTIC mapping, the following key assumptions were made: (i) Contamination will occur at the ground surface; (ii) the contaminant enters the water table when rain falls on the surface and percolates into the saturated zone; (iii) the contaminant travels with water, at the same rate as water; and (iv) the shallow aquifers are unconfined. Each DRASTIC parameter mapping was assigned a rating and a weight, as stated by Aller et al. [11], as follows:

DRASTIC Index (DI) = D_R_D_W_ + R_R_R_W_ + A_R_A_W_ + S_R_S_W_ + T_R_T_W_ + I_R_I_W_ + C_R_C_W,_

where D, R, A, S, T, I, and C are the seven parameters of the model, and the subscripts R and W are the corresponding ratings and weights, respectively. This was calculated for the 61 boreholes used in this study. The rating and weight is shown in Table 2. For example, the DI was derived from the assigned weight of the parameters, such as 5 for depth to water, multiplied by the ratings which were based on the range of how deep the water level was. 

### 2.1. DRASTIC Parameters 

The depth to water table of shallow wells was measured in the field and contoured by gridding using the Windows Interpretation System for Hydrogeologist (WISH) software, version 3.0.2.187. WISH is compatible and similar to geographical information system (GIS) software used in most vulnerability mappings [27,28,39]. Weights assigned by DRASTIC to each mapping parameter are shown in Table 2. The highest weight of 5 was assigned by DRASTIC to the depth to water table and the impact of the vadose zone. To calculate the net recharge for the DRASTIC mapping, evapo-transpiration (ETR) and run-off values from previous studies in the basin were used (Table 3). ETR in the Dahomey Basin has substantial significance on the recharge rate due to its climatic location, the tropical rainforest belts. The Blaney Morin Nigeria and Priestly Taylor methods were used to calculate the ETR and run-off for Abeokuta, Lagos, and Ijebu-Ode [40,41]. The net recharge was calculated using the formula:

Net recharge = [rainfall − (evapo-transpiration + run-off)] × recharge rate

Twenty years’ rainfall data sourced from the Nigerian Meteorological Agency shows a mean rainfall pattern of 1200 mm/year for the northern part of the Dahomey Basin, and 1800 mm/year for the southern part of the Basin. Rainfall serves as the basic mode of groundwater recharge [42] in the basin. The rainfall is influenced by the Inter-tropical Convergence Zone (ITZC) wind, which blows from the Atlantic Ocean to the Sahara Desert. ITZC is also responsible for the seasonality of the rainfall and the two rainfall peaks in June and September. A dry spell is noticed in August and is below 100 mm in Lagos [40] and 88.3 mm in Abeokuta [41]. Groundwater level is influenced by the seasonality of the rainfall. Authors in [1] reported a fluctuation of 5 m for the groundwater of Lagos, which was primarily due to the rainy and dry seasons. 

The important parameter mapped for the aquifer media was the aquifer bed characteristics, their hydraulic properties, and the attenuation capacity. Lithology and sediment were interpreted from the geological map of Nigeria, field hydro census, and well log data. Aquifer media were assigned a rating of six for both fine to medium sand, and a rating of eight for coarse sand (Table 2). Often, the aquifer media material is the same as the vadose material presented in the DRASTIC methodology [11]. Soil media was derived from the plot of the particle size analysis. The result was compared to well log data. The top soils and subsoils of the basin were prioritised to derive the soil media. This was to avoid examining the same material for soil media and aquifer media, particularly in areas of the shallow water table.

Topography controls the likelihood of contaminant infiltration and it is calculated from the slope. The slope was derived from the digital elevation model (DEM) and topographic map. A DEM image extracted from the National Aeronautics and Space Administration (NASA) and the United States Geological Survey (USGS) Landsat imageries was the basis upon which surface elevation was delineated. The elevation model was reprocessed from the Global Land Survey (GLS) collection. The GLS collection contained imageries from TM, ETM+, and ALI sensors. GLS–DEM uses a 90 m resolution and covers 185 km × 185 km. This translates to one-degree latitude by one-degree longitude. Anomalies in the DEM images were corrected with ground surface GPS readings. The slope was further crosschecked with the topographical map of the basin. 

The vadose zone’s influence on aquifer pollution potential was essentially like that of soil cover, depending on its permeability and on the attenuation characteristics of the media. The impact of the vadose zone was prepared from the geological map of Nigeria on a scale of 1:50,000 and examined borehole logs. The hydraulic conductivity values of the different sedimentary formation areas were collated from drillers’ pump test data, and borehole reports from the Nigeria Geological Survey [37]. A major flaw of the DRASTIC method is the difficulty in calculating an accurate value for hydraulic conductivity [39]. The Dahomey Basin’s hydraulic conductivity varies from 69,158 to 13,726 gallons per day (gpd) for the Ilaro Formation aquifers, 25,973 to 31,193 gpd for the Alluvium aquifers, 12,680 to 34,870 gpd for the Coastal Plain Sand, and 1014 gpd for a major part of the Abeokuta Formations.

### 2.2. Sensitivity Analysis

Sensitivity analyses are used to establish the relationship between the DRASTIC mapping parameters [17,43]. The DRASTIC method is characterised by using a high number of parameters, which is believed to limit the impact of errors and uncertainties in the individual parameters on the final outputs [12,17,44]. There are two types of sensitivity analyses—single-parameter sensitivity and map removal sensitivity. The single-parameter sensitivity analysis, designed by [45], aimed to compare the subjectivity in assigning weight and scores to the DRASTIC theoretical weight and the effective weight, and is calculated as follows:

$$W=100P_{r}P_{w}/V$$

where *W* refers to the effective weight of each parameter, *P_r_* and *P_w_* are the ratings value and the weight of each parameter, respectively, and *V* is the overall vulnerability index.

Map removal sensitivity identifies the sensitivity of the vulnerability towards removing one or more maps from the vulnerability analysis [46]. It is computed as follows:

$$S=\left(\frac{\left|\frac{V}{N}-\frac{V^{\prime }}{n}\right|}{V}\right)\times 100,$$

where *S* = the sensitivity measure expressed as variation index, *V* and *V*′ = the unperturbed and perturbed vulnerability indices, respectively, and *N* and *n* = the number of parameters used to compute *V* and *V*′, respectively. The unperturbed indices are the values derived from the seven vulnerability mapping parameters, while the perturbed vulnerability indices are acquired using a lower number of parameters.

## 3. Results and Discussion

### 3.1. DRASTIC Vulnerability Parameters

The results of the seven DRASTIC parameter mappings are presented in sequential order. Evaluation of each parameter is based on salient properties of the aquifer, overlying lithology, climatic conditions, and visual observation around the study area. 

### 3.2. Depth-to-Water Table

For shallow aquifers, the depth to water is the distance to be covered from the point on the land surface to groundwater table. Depth to water table is an important factor because it determines the thickness of the material through which infiltrating water must travel before reaching the saturated zone. The deeper the water table, the lesser the chances of pollutants entering groundwater and the less likely the transportation of contaminants within the aquifer. Filtration and degradation of contaminant introduced at the surface increases with increasing travel time of contaminant. Therefore, the higher the depth to water table, the higher the possibility of degradation of the percolating contaminant. A summary of the depth to water DRASTIC index derived in the Dahomey Basin from the 56 mapping data points covering the entire basin is presented in Table 2. In general, the shallow aquifer potential protection increases with the depth to water table. This means that the areas with depth to water table >31 m will have less potential to contamination compared to areas with a depth to water table below 5 m. 

### 3.3. Net Recharge

**Figure 2:**
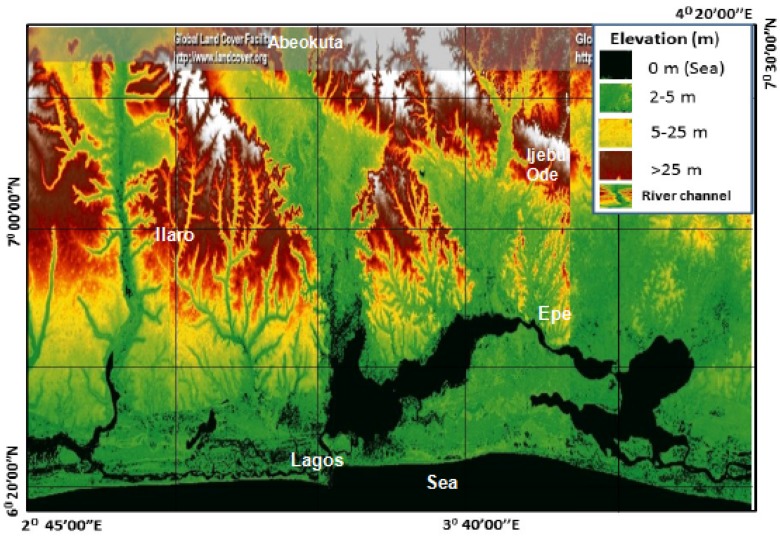
Digital Elevation Model (DEM) of the Dahomey Basin showing surface water bodies in black.

**Figure 3:**
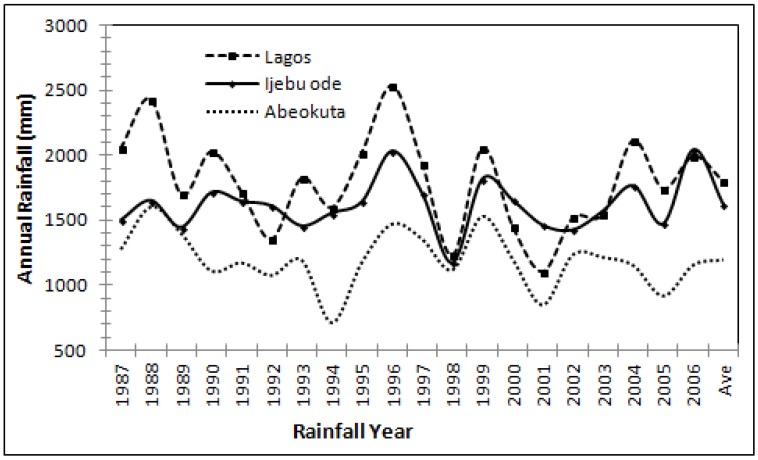
Annual rainfall of three areas of the Dahomey Basin.

Net recharge in DRASTIC is the summation of available water that has the potential of reaching groundwater. The water source includes precipitation and other water sources. Precipitation is considered as the driving force because it has the potential to initiate percolation and transport contaminants within the vadose zones to the saturated zones. Precipitation has the capacity to carry the solid and liquid contaminants to the water table. Most of the southern end is associated with ponding and creeks (Figure 2), particularly the southeastern part with a DRASTIC index of 40. The northern end of the Dahomey basin (Abeokuta) where rainfall is the lowest (Figure 3) presents the least DRASTIC index score of 4. The northern areas of the basin have high runoff due to the undulating topography, and recharge is lowest (Table 3). Table 4 shows the net recharge and rating derived from the calculation in Table 3. 

**Table 3:** Net recharges estimation from precipitation and runoff (mm/year).

Parameters	Lagos	Ijebu-Ode	Abeokuta
Rainfall (mm)	1800	1600	1200
ETR (mm)	1367 ^1^	1276.0 ^2^	1133 ^2^
Run-off (mm)	352 ^3^	48.6	48.6 ^4^
Total recharge (mm)	81	276	18.4

^1^ [40].^2^ [41].^3^ [47].^4^ [48].

### 3.4. Aquifer Media

The unconsolidated sedimentary rocks are the aquifer media in Dahomey Basin. The shallow unconfined aquifers of these rocks were targeted for mapping since it is the most accessible to the local populace and most susceptible to contamination due to the short travel distance of contaminants introduced from the surface. The aquifer media includes limestone, sandstone, alluvium, and sandy clay. In unconsolidated aquifers, the attenuation is based on the sorting and amount of fine materials within the aquifer. The shallow aquifers occur within a depth of <3 m along the coastal areas and wetland of the River Ogun to 45 m in the Abeokuta. These areas contain sand and gravel, and recorded the highest DRASTIC index of 24–25. The larger the grain sizes, the higher the permeability, thus the vulnerability of the aquifer. 

### 3.5. Soil Media

Soil and its different media is an important component in the DRASTIC formulation. Filtration, degradation, and disintegration of heavy contaminants starts from soil. These complex processes are determined by the amount of fine materials in soil, especially clay and silt. Soils with high fine component have better filtration and degradation capacity. Therefore, contaminant attenuation increases from very coarse media to fine media. Other important soil media property that reduces shallow groundwater vulnerabilities includes porosity, texture, clay types, and grain sizes. The Dahomey soil media includes sandstone, alluvium, sandy loam, and loam. The DRASTIC method rates loam with respect to sandy and gravely soil types. Sandstone soil media have higher DRASTIC index rates (18) as compared to alluvium (14) in the soil media (Table 2). These areas have poor attenuations to contaminant. The presence of fine grain materials, such as clay and silt represented by loam, presents the lowest DRASTIC index of 10 in the soil media, and the percentage of organic matter within the soil cover can decrease intrinsic permeability. This can retard or prevent contaminant migration via physico-chemical processes, namely absorption, ionic exchange, oxidation, and biodegradation [16]. 

### 3.6. Topography

Topography refers to the slope of the land surface. The slope varies from the upper end of the basin (>18 m) and decreases towards the sea (3–4 m). This implies that the land surface in the basin generally slopes gently downwards from north to south, with the DRASTIC index increasing from 1 to 8, respectively (Table 2). Water is the most significant topographical feature in the southern end of the basin, particularly in the Lagos area, where water and wetlands cover over 40% of the total land area within the State. Iwugo et al. [49] reported that aside areas of wetlands, an additional 12% of the land are subjected to seasonal flooding. The Atlantic Ocean impacts the topography greatly. All adjacent areas along the coast are relatively flat (Figure 2). Other physical features which have an impact on the basin slopes are Lagos Lagoon, Ogun River, and the Ewekoro River. Flat land, swamps, and coastal areas of the Atlantic Ocean record slope ranges of 3–5 m (Table 2). Flat areas were assigned high vulnerability rates because their run-off rate was less, so more percolation of contaminants to the groundwater was expected. The highest DRASTIC index of 8 from the topography on the Dahomey Basin reflects how topography has a low to moderate effect on groundwater vulnerability [50]. 

### 3.7. Impact of Vadose Zone

**Figure 4:**
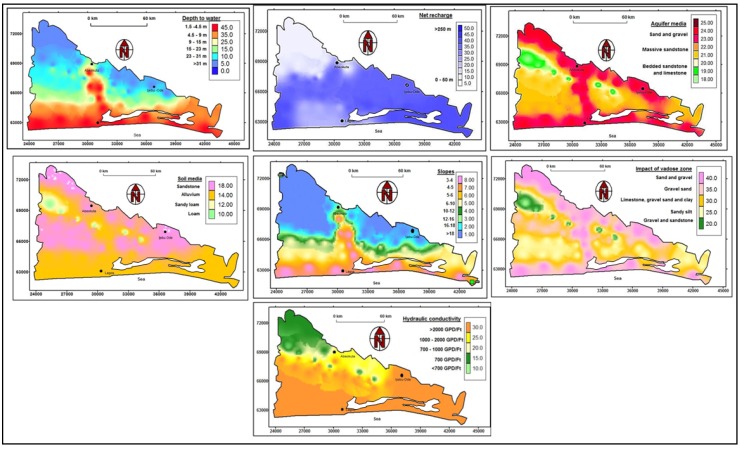
Maps of the seven DRASTIC parameters.

The vadose zone has a high impact on water movement if it is composed of impermeable material. Areas with sand and gravel scored the highest DRASTIC index of 40 (Table 2), indicating its high influence on aquifer vulnerability. These are areas along the coast, wetlands, and river channels in the basin and the northern areas of the basin where sedimentary rocks overlie the Basement rocks of southwestern Nigeria. Limestone occurrence areas scored a DRASTIC index of 30, showing its moderate vulnerability impact. The larger areas of the basin DRASTIC index scores varied from 25–30, indicating a moderate impact on the shallow aquifer. The moderate impact is expected to significantly impact the overall vulnerability map. The moderate impact areas were covered by sandy silt, gravely sand, and some clay. Table 2 shows the weights and ratings of the vadose zone, and the map is shown in Figure 4. 

### 3.8. Hydraulic Conductivity

Aquifer hydraulic conductivity depends on the intrinsic permeability of the material and on the degree of saturation of the aquifer media. This critical factor controls the contaminant migration and dispersion from the injection point within the saturated zone. The coastlines of the Atlantic Ocean and larger part of the basin hydraulic conductivities were above 2000 gpd, and their DRASTIC index was 30. This suggests high vulnerability to contamination because an aquifer with high hydraulic conductivity is vulnerable to substantial contamination, as a plume of contamination can move easily through the aquifer [16]. The DRASTIC index value increased from 15 to 25 from the southern end to the northern end of the basin, where vulnerability is expected to reduce due to low conductivity.

### 3.9. DRASTIC Map

**Figure 5:**
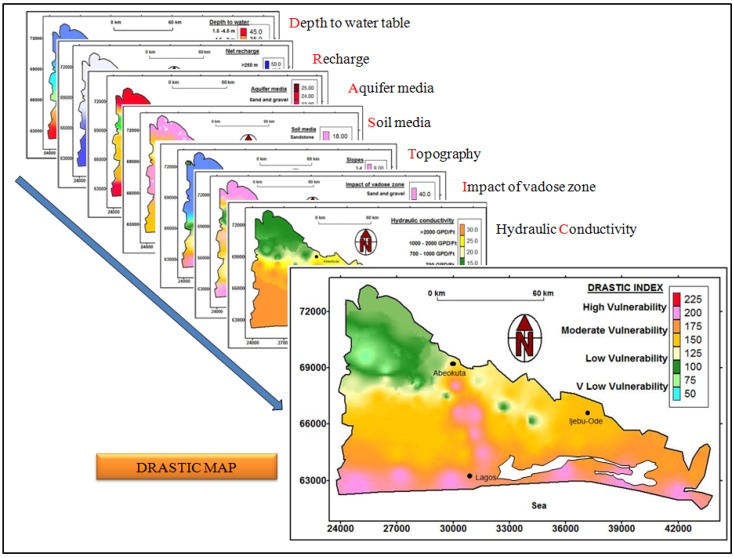
DRASTIC vulnerability maps of Dahomey Basin.

The resultant DRASTIC map produced from interpolation and direct weighting of the seven DRASTIC parameters is shown in Figure 5. The DRASTIC index model by [11] suggests that the minimum vulnerability value is 24 and the maximum is 220. Dividing this range into four equal classes gives a class range of 24–71 (very low or no risk), 72–121 (low), 122–170 (moderate), and 172–220 (high vulnerability risk). Therefore, the results of DRASTIC vulnerability values in the basin lay between 44 and 210 (Figure 5). This ranges from very low to high vulnerability. 

The DRASTIC vulnerability distribution of the Dahomey Basin shows high vulnerability for areas with low depth-to-water, high rainfall, and flat to low topography. These areas include the coastlines of the Atlantic Ocean and wetlands along the Ogun River, which cover 21% of the basin. The very low to low vulnerability was mapped for areas that had high vadose thickness, likely run-off, and the lowest rainfall section of the basin. Other intrinsic parameters contributing to low or very low vulnerability mappings are a high slope and depth to water table, and compacted soils and rock type. The low to very low vulnerability areas covered 18% of the Dahomey Basin. The majority of the areas in the basin are classified as moderate vulnerability with a value of 61%. These areas cover a large part of the Lagos and Ogun metropolis. The average depth to water table is 21 m in these areas. 

### 3.10. Sensitivity of the Map

The best way to deal with subjectivity associated with the DRASTIC maps is to carry out a sensitivity analysis. Table 5 presents a statistical summary of the seven rated DRASTIC mapping parameters. Fifty-six boreholes were used for these statistical purposes. The highest risk to groundwater contamination was from the impact of the vadose zone (mean: 31.6), net recharge (mean: 30), hydraulic conductivity (mean: 26.7), and to a lesser extent, from depth to water (mean: 21.6), aquifer media (mean: 21), and soil media (mean: 15.5). Topography (slope) with a mean of 3.6 presented the lowest risk to contamination among the mapping parameters. Topography is reported to have the lowest impact on DRASTIC vulnerability [50] because it is assigned the lowest weight. The variations coefficient (C_V_) among the aquifer media parameter was the highest (C_V_: 91%), and lowest in hydraulic conductivity (C_V_: 23.6%). The highest variation was due to the style of rating of the rock type in DRASTIC parameters. Rock types of massive sandstone and the bedded sandstones ratings were different. Subsequently, this was rated differently to sand with gravel. However, the Dahomey aquifer media parameter records the lowest standard deviation, which is a sign of uniformity of sandstone in this case. 

### 3.11. Map Removal Sensitivity 

Map remove sensitivity is done by removing one or more data layers. Map removal takes into account all the well data points used in computing the DRASTIC map. It is observed that the map sensitivity is the highest upon removal of topography, with highest mean variation (13.9). This is attributed to the sharp differences in slope variation prevailing in the basin. The Dahomey Basin coastal land elevations were close to the sea’s elevation. These contrast sharply in most northern areas of the Dahomey Basin which are closer to the Basement rocks. The map is least sensitive to the impact of the vadose zone (10.7). This results from the uniform composition of the Dahomey sedimentary formations, which are largely alluvium and sandstones. Other parameters of the map are sensitive to the depth to water table and the soil media, as shown in Table 6. 

### 3.12. Single-Parameter Sensitivity 

The benefit of the DRASTIC vulnerability mapping is the high significance number of parameters used in its computation. The single-parameter removal sensitivity analysis test indicated the influence of each parameter on the final vulnerability measurement [27]. The weight assigned to these parameters required sensitivity analysis in order to compare the effective weight from the theoretical weight, as proposed in the DRASTIC method. Table 7 shows the result of the single-parameter sensitivity analysis. The effective weight is a function of the actual value of a single parameter, as compared to the other six parameters, as well as to the weight assigned to it by the DRASTIC method (12, 16]. This means that the effective weight should have been the actual weight of the DRASTIC methodology. It is observed from Table 7 that DRASTIC assigned too much importance to depth to water with (21.7%), while its effective weight is (13%). These results differ from [50], where they considered the vadose zone and aquifer media’s effective weight to be higher than the theoretical weight in their DRASTIC application. Topography, soil media, aquifer media, and net recharge were undervalued by the DRASTIC method. The result of the single parameters’ sensitivity shows the importance of the mapping parameters as follows, I>R>C>A>D>S>T, against the theoretical weight DRASTIC method, in the order of D>I>R>A>C>S>T.

### 3.13. Parameter Correlation

DRASTIC parameters’ correlation is another way to establish the interdependency or replication of DRASTIC parameters employed in vulnerability mapping. Comparison correlation is one of the best methods of comparing vulnerability results [51]. Table 8 shows the DRASTIC correlation matrix values of the Dahomey Basin. Nearly perfect correlation (0.92) is between the depth to water and topography. This is due to the similarity of their ratings. However, their weights are different with values of 5 and 1, respectively. Furthermore, a depth to water and topographic correlation significance level of 1.6 × 10^−23^ suggest the correlation is highly significant. It must be noted that the near perfect correlation is susceptible to drastic changes on the field due to human-induced factors, such as land use and excessive pumping, which controls the water level fluctuations. Aquifer media and the impact of vadose zone correlations of 0.89 suggest the impacts of replication of the parameters in DRASTIC, especially when the correlation significance level is 2.7 × 10^−20^. This is evidenced in Table 2. This possibility has been suggested by [52] and [53]. Soil media record a negative correlation with many other parameters (topography, net recharge, and depth to water). Recharge is least correlated with vadose zone and soil media. This means net recharge is an independent variable and effective parameter in DRASTIC vulnerability evaluation.

The Dahomey Basin is a coastal basin with a high population density. The high urban densities suggest an increase in health risks due to groundwater contamination. The shallow groundwater of the coastal areas presents the highest risk to contamination in the basin. This is due to the coastal areas’ low intrinsic property derived from the high DRASTIC indices. Part of this coastal area has been classified as highly vulnerable and polluted [33]. Unlike the low vulnerability areas which have a greater potential of degrading contaminants flowing to the shallow aquifer due to low DRASTIC indices, the coastal areas’ water table is the lowest in the basin. It also shows the areas which have the highest recharge, unconsolidated vadose materials with a flat topography at par with the sea level.

## 4. Conclusions

The unprecedented population rise in the Dahomey Basin and risk of groundwater contamination from over-population to the basin’s shallow aquifers underlies the importance of assessing the intrinsic vulnerability of the basin. The DRASTIC vulnerability model was used to express the intrinsic vulnerability property of the aquifer in the event of pollution. DRASTIC employed seven parameters for its vulnerability mapping and evaluations. The investigation classifies shallow groundwater in the Dahomey Basin into high, moderate, and low to very low vulnerability areas, and the risk involved if the vulnerable groundwater in the basin remains unprotected. Special attention and laws regulating land use activities are needed for areas shown to be more vulnerable to pollution. Other less vulnerable areas can be a site for landfill and groundwater polluting activities.

The impact of the vadose zone and recharge has shown to be a major relevant parameter that must be considered by users of the DRASTIC method for intrinsic vulnerability assessment and the risk of an aquifer to pollution. It is important to address recharge and the impact of vadose parameters because they show the greatest risk for shallow groundwater assessment using the DRASTIC method [13,54]. Recharge to an aquifer is one major path through which contaminants are transported to an aquifer. The mode and degree of groundwater recharge is highly connected to land use. However, the land use parameter is not part of DRASTIC and was not assessed in this study due to a lack of data, and is suggested to be added to any further studies. This is a weak point in this assessment, particularly from the health risk perspective. In order to prevent groundwater from contamination and its associated health risks, intrinsic vulnerability mapping is an important factor for providing a solution to groundwater protection and preventing spread of diseases associated with water usage. At present, there are no laws in Nigeria protecting vulnerable groundwater areas. Mapping these intrinsic vulnerability properties of the Dahomey Basin using the DRASTIC method should guide in formulating one and to prioritise areas shown in the study to be most vulnerable to surface contaminations.

A major significant point highlighted in the study included showing the sensitivity of the basin vulnerability map to the DRASTIC vulnerability parameters. Topography shows the least parameter of the intrinsic property influencing the vulnerability map, while the vadose zone property shows the highest parameter. The single parameters’ removal sensitivity analysis indicated the order in which single parameters affected the final intrinsic vulnerability of the Dahomey Basin as an impact of the vadose zone, net recharge, conductivity, aquifer media, depth to water table, soil media, and topography, respectively. The usage of the vulnerability map as presented in the study should be used with some restraint, especially considering the health impacts of untested water quality, even though careful field procedures and investigations were undertaken to produce the vulnerability map of the basin. This can be addressed with further ground investigation of water qualities. The maps strictly accounted for the intrinsic properties of the local regional aquifer of the Dahomey Basin, and its overlying beds and abilities to filter and degrade contaminated water. Their use was intended for planners, decision makers, and as education tools. DRASTIC applications to pollution, as applied in this context, guides against environmental health disasters that might occur due to pollution occurrence. It is a means of preventing pollution, rather than containment of pollution.

## Figures and Tables

**Table 1:** Sources of data employed in the DRASTIC computation.

Parameters	Description	Source
Depth–to–water	Represents the depth from the ground surface to the water table. Deeper water table implies lesser chance for pollution to occur.	Data were generated from the study area and from local drillers’ directories.
Net recharge	Represents the amount of water that penetrates the vadose zone and reaches the water table. Recharge water represents the vehicle for transporting pollutants.	Generated from rainfall data from Nigeria’s Metrological Agency and previous calculated evaporation and run-off.
Aquifer media	Refers to the saturated zone material properties, which controls the pollutants’ attenuation processes.	Field studies and interpretation of geological map of Nigeria on scale 1:50,000.
Soil media	Represents the uppermost weathered portion of the vadose zone and controls the amount of recharge that can infiltrate downwards.	Generated from field and laboratory studies.
Topography	Refers to the slope of the land surface. It indicates whether the run-off will remain on the surface to allow pollutant percolation to the saturated zone.	Digital Elevation Model (DEM) of the basin available at the Global Land Cover Facility (GLCF) of Maryland University and topography map.
Impact of vadose zone	This is defined by the vadose zone material, which controls the passage and attenuation of the contaminated material to the saturated zone. The vadose zone and aquifer media are the same materials.	Interpretation of the geological map of Nigeria from NGSA.
Hydraulic conductivity	Indicates the ability of the aquifer to transmit water, thus determining the rate of flow of the contaminants within the ground water system.	Derived from previous literature as well as reported drillers’ records.

**Table 2:** Dahomey Basin DRASTIC parameters and DRASTIC Index (DI).

**Depth-to-water × 5**	**DRASTIC rating**	**DI**
1.5–4.5 m	9	45
4.5–9 m	7	35
9–15 m	5	25
15–23 m	3	15
23–31 m	2	10
>31 m	1	5
**Net recharge × 4**	**Rating**	**DI**
>250 mm/y	10	40
50–100 mm/y	3	12
0–50 mm/y	1	4
**Aquifer media × 3**	**Rating**	**DI**
Sand and gravel	8	24
Massive sandstone	7	21
Bedded sandstone and limestone	6	18
**Soil media × 2**	**Rating**	**DI**
Sandstone	9	18
Alluvium	7	14
Sandy loam	6	12
Loam	5	10
**Topography × 1**	**Rating**	**DI**
3–4 m	8	8
4–5 m	7	7
5–6 m	6	6
6–10 m	5	5
10–12 m	4	4
12–16 m	3	3
16–18 m	2	2
>18 m	1	1
**Impact of vadose zone × 5**	**Rating**	**DI**
Sand and gravel	8	40
Gravel sand	7	35
Limestone, gravel sand and clay	6	30
Sandy silt	5	25
Gravel and sandstone	4	20
**Hydraulic conductivity × 3**	**Rating**	**DI**
>2000 gpd/ft^2^	10	30
1000–2000 gpd/ft^2^	8	24

**Table 4:** Rating of net recharge for the Dahomey Basin.

Location	Dahomey Recharge	DRASTIC Rating	DI × 4
Lagos	81	5	20
Ijebu-Ode	276	10	40
Abeokuta	18.4	2	8

**Table 5:** Statistics of the DRASTIC parameters map.

Descriptive Statistics	Parameters						
	D	R	A	S	T	I	C
Min	5	8	18	10	1	15	12
Max	45	40	24	18	8	40	30
Mean	21.6	30	21	15.5	3.6	31.6	26.7
SD	15.7	12	1.9	2.7	2.8	8.4	6.3
C_V_	72.7%	40%	91%	17.4%	77.8%	26.6%	23.6%

**Table 6:** Statistics of map removal sensitivity.

	Co-Efficient of Variation				
Parameters	Mean	Min	Max	SD	C_V_
D	12.12	10.14	13.74	1.26	10.4%
R	11.01	8.10	13.24	1.25	11.3%
A	11.76	9.94	12.29	0.61	5.1%
S	12.48	11.58	13.12	0.46	3.6%
T	13.92	13.55	14.18	0.24	1.7%
I	10.73	8.28	11.83	0.93	8.6%
C	11.31	10.22	12.22	0.48	4.2%

**Table 7:** Statistics of the single parameters’ sensitivity analysis.

			Effective Weight			
Parameters	DRASTIC Weight	% DRASTIC Weight	Mean	Min	Max	SD
D	5	21.7	13	3.2	24.8	7.5
R	4	17.4	19.7	6.2	37.1	7.5
A	3	13	15.2	11.9	26	3.6
S	2	8.7	10.8	6.9	16.2	2.7
T	1	4.3	2.2	0.6	4.4	1.4
I	5	21.7	21.5	14.7	36	5.5
C	3	13	17.8	12.3	24.3	2.9

**Table 8:** Pairwise correlations matrix of the DRASTIC parameters.

Parameters	Mean	SD	D	R	A	S	T	I	C
D	13.01	7.57	1.00						
R	19.74	7.51	0.38	1.00					
A	15.24	3.68	0.45	0.14	1.00				
S	10.86	2.80	−0.35	−0.02	0.34	1.00			
T	2.21	1.42	0.92	0.43	0.47	−0.30	1.00		
I	21.50	5.54	0.27	0.06	0.89	0.52	0.19	1.00	
C	17.88	2.91	0.52	0.47	0.39	0.49	0.45	0.44	1.00
